# Study on inflammation and fibrogenesis in MAFLD from 2000 to 2022: a bibliometric analysis

**DOI:** 10.3389/fendo.2023.1231520

**Published:** 2023-08-31

**Authors:** Kuanhong Luo, Yang Chen, Shuzheng Fang, Siqi Wang, Zhixin Wu, Huiqing Li

**Affiliations:** ^1^ Department of Endocrinology, Union Hospital, Tongji Medical College, Huazhong University of Science and Technology, Wuhan, China; ^2^ College of Art and Sciences, Washington University in St. Louis, St. Louis, MO, United States

**Keywords:** inflammation, fibrosis, MAFLD, bibliometric analysis, NAFLD

## Abstract

Chronic inflammation and fibrosis are significant factors in the pathogenesis of metabolic-associated fatty liver disease (MAFLD). In this study, we conducted a bibliometric analysis of publications on inflammation and fibrogenesis in MAFLD, with a focus on reporting publication trends. Our findings indicate that the USA and China are the most productive countries in the field, with the University of California San Diego being the most productive institution. Over the past 23 years, Prof. Diehl AM has published 25 articles that significantly contributed to the research community. Notably, the research focus of the field has shifted from morbid obesity and adiponectin to metabolic syndrome, genetics, and microbiome. Our study provides a comprehensive and objective summary of the historical characteristics of research on inflammation and fibrogenesis in MAFLD, which will be of interest to scientific researchers in this field.

## Introduction

MAFLD, formerly known as non-alcoholic fatty liver disease (NAFLD), is distinguished by hepatic steatosis and the presence of one or more of the following factors: overweight/obesity, type 2 diabetes mellitus (T2DM), or indications of metabolic dysregulation (1). Strikingly, MAFLD affects approximately a quarter of the population worldwide with a rising trend (2) and involves a spectrum of liver diseases that range from simple steatosis to its progressive form, non-alcoholic steatohepatitis (NASH), characterized by inflammation and progressive tissue fibrosis, and may lead to the development of cirrhosis and even hepatocellular carcinoma (HCC) (2). Moreover, MAFLD aggravates the deteriorative progression of T2DM and its complications (3), and significantly increases the risk of chronic kidney disease (CKD) (4) along with cardiovascular disease (5). Nevertheless, the clinical management of MAFLD is currently restricted to lifestyle interventions with no approved drug therapy for the disease.

It has been widely accepted that chronic inflammation and fibrosis formation play pivotal roles in the pathogenesis of MAFLD (6). In a physiological state, the hepatic inflammatory response is a response to various stress conditions, which is beneficial to repairing tissue damage and promoting hepatic homeostasis (7). However, prolonged or intense inflammatory reactions may result in irreversible liver damage, such as liver fibrosis, which is triggered by the activation of hepatic stellate cells (HSCs) and their transdifferentiation into myofibroblasts (8). According to the “multiple hit” hypothesis, a comprehensive and detailed theory focusing on the pathomechanism of MAFLD, inflammation may precede steatosis in NASH, and inflammatory mechanisms are involved in the entire process of MAFLD (9). Moreover, synergistic effects of pathological events, such as endoplasmic reticulum (ER) stress (10), insulin resistance (11), aberrant lipid metabolism (12), oxidative stress (13), and mitochondrial dysfunction (14), make great contributions to the exacerbation of inflammation and the deterioration of fibrogenesis *via* various pathways, and have all been implicated in the progression of MAFLD. Therefore, it is meaningful to illustrate and summarize the research trend on inflammation and fibrogenesis in MAFLD with the hope of discovering drug targets and developing effective therapies.

Comparatively to literature reviews, bibliometrics conducts quantitative research on the field’s literature by analyzing its characteristics with the help of visualizing processing tools, like CiteSpace or VOSviewer, to identify the predominant institutions/countries, leading authors and journals, top-cited references, research trend, or hotspots (15). Considering that hepatic inflammation and fibrogenesis are of great importance in the progress of MAFLD, no bibliometric study has been reported on this topic. Therefore, our study aims to identify the publication trends and potentially significant hotspots on inflammation and fibrogenesis in MAFLD by analyzing the records published from 2000 to 2022.

## Materials and methods

### Data sources and search strategies

**Figure 1:**
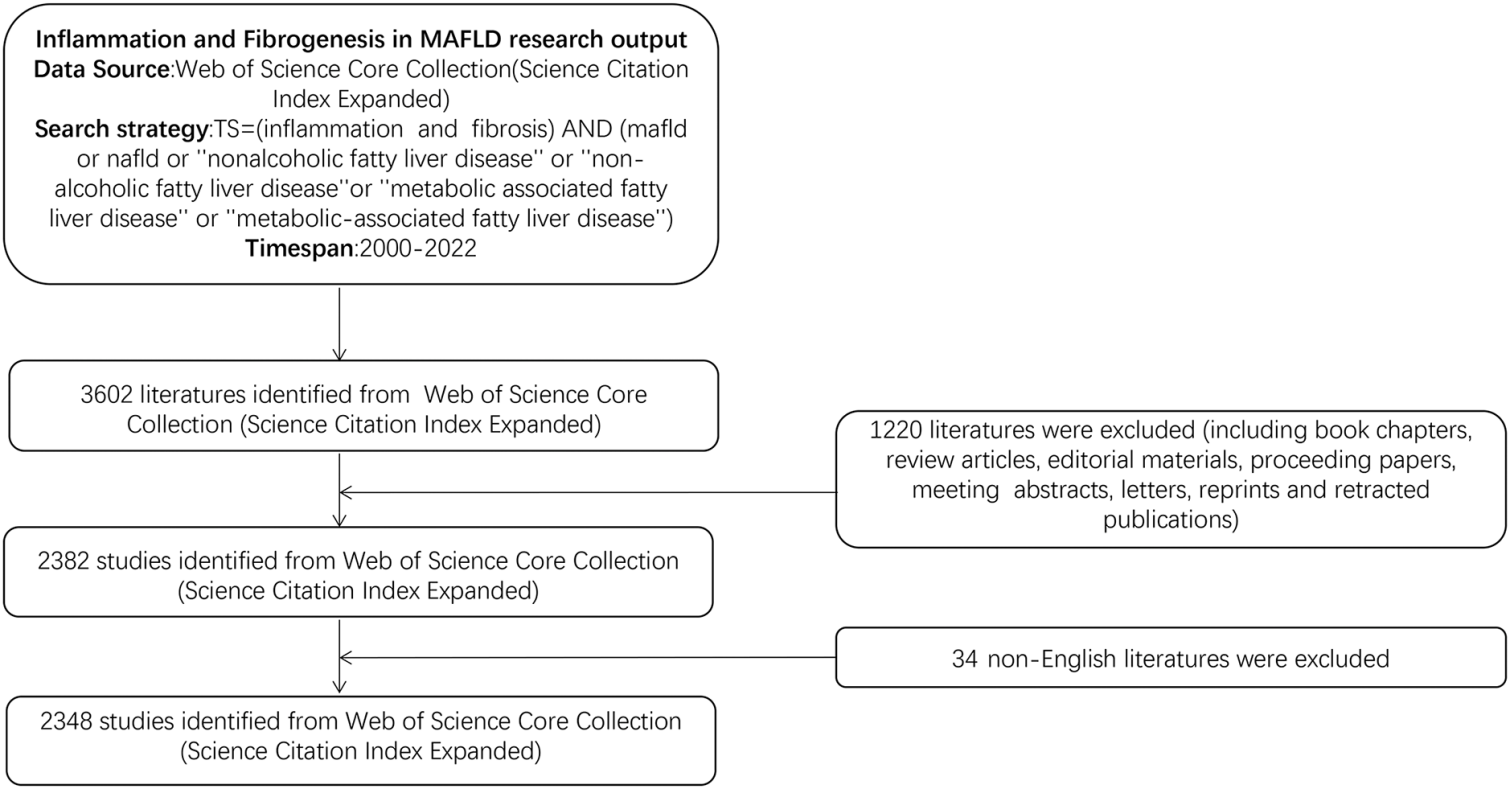
Flowchart of this study.

In our study, we selected the Web of Science Core Collection Science Citation Index Expanded (WoSCC-SCIE) database for the literature search from 2000 to 2022 on 6 January 2023. All data extraction and downloads were completed on the same day to avoid bias in database updates. The search strategy is as follows: (TS = (inflammation and fibrosis)) AND (TS = (mafld or nafld or ‘‘nonalcoholic fatty liver disease’’ or ‘‘non-alcoholic fatty liver disease’’ or ‘‘metabolic associated fatty liver disease’’ or ‘‘metabolic-associated fatty liver disease’’)), then non-English literature and other types of literature were excluded, and only articles were enrolled in this study. Then, the raw data were downloaded from WoSCC-SCIE as text files involving full records and cited references. A total of 2,348 articles were ultimately analyzed in our study. The detailed flowchart is shown in **Figure 1**.

### Bibliometric analysis

We mainly analyzed the data by VOSviewer (version 1.6.18) and CiteSpace software (version 6.2.R2 advance). The specific method was described before (16). VOSviewer is a software tool for constructing and visualizing bibliometric networks, and is often used to summarize the most prolific countries/regions, institutions, journals, and authors (see www.vosviewer.com). In our study, VOSviewer was used to show the top 10 most cited journals and achievements of different countries/regions and institutions. CiteSpace is another metrological analysis software developed by Prof. Chen C for bibliometric analysis and visualization (17). In this study, CiteSpace was used to evaluate multiple indicators, including the collaboration between countries/regions and authors, co-citation analysis, citation burst, clustered networks of co-cited references, and keywords with the strongest citation bursts.

## Results

### Quantity and trends analysis of published papers

**Figure 2:**
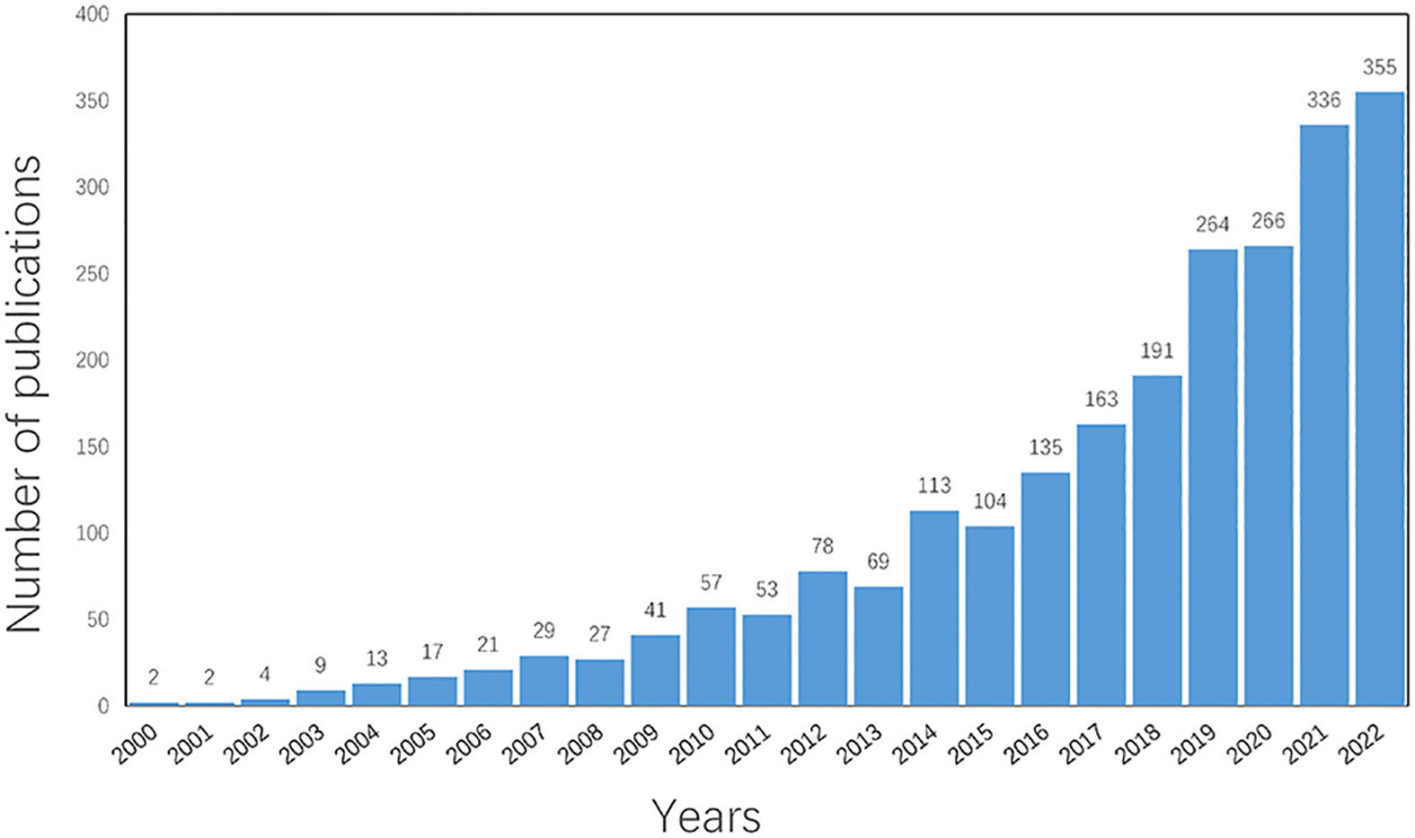
Tthe number of publication from 2000 to 2022.

A total of 2,348 documents were retrieved from the WoSCC-SCIE databases between 2000 and 2022 according to the flowchart shown in **Figure 1**. As shown in **Figure 2**, there was an overall upward trend in the amount of literature on inflammation and fibrogenesis in MAFLD, although in some years, the amount of literature could be declining. It is worth noting that 1,412 articles were published in the past 5 years, accounting for 60.14% of the total, implying that MAFLD has become a common chronic disease that has attracted the attention of researchers worldwide.

### Productive countries/regions and institutions

To determine which countries or regions have contributed the most to the development of this field during the past 23 years, we counted the number of articles published by different countries and regions using VOSviewer, and the top 10 most productive countries or regions are shown in **Table 1**. We found that the USA ranked first, followed by China, Japan, Italy, and Germany. Meanwhile, we concluded that the number of publications from the USA and China far exceeded those other countries/regions. In addition, the number of publications in China first surpassed that in the USA in 2021.

**Table 1:** The top 10 productive countries and institutions in research of inflammation and fibrosis in MAFLD from 2000 to 2022.

Rank	Country	Count	Rank	Institution (country)	Count
1	USA	704	1	Univ of California San Diego (USA)	74
2	China	516	2	Duke Univ (USA)	43
3	Japan	265	3	Shanghai Jiao Tong Univ (China)	41
4	Italy	173	4	Mayo Clin (USA)	37
5	Germany	169	5	Harvard Med Sch (USA)	35
6	South Korea	123	6	Virginia Commonwealth Univ (USA)	35
7	England	117	7	Nci (USA)	33
8	Spain	102	8	Univ Milan (Italy)	32
9	France	94	9	Wenzhou Med Univ (China)	31
10	Australia	75	10	Univ Washington (USA)	30

**Figure 3:**
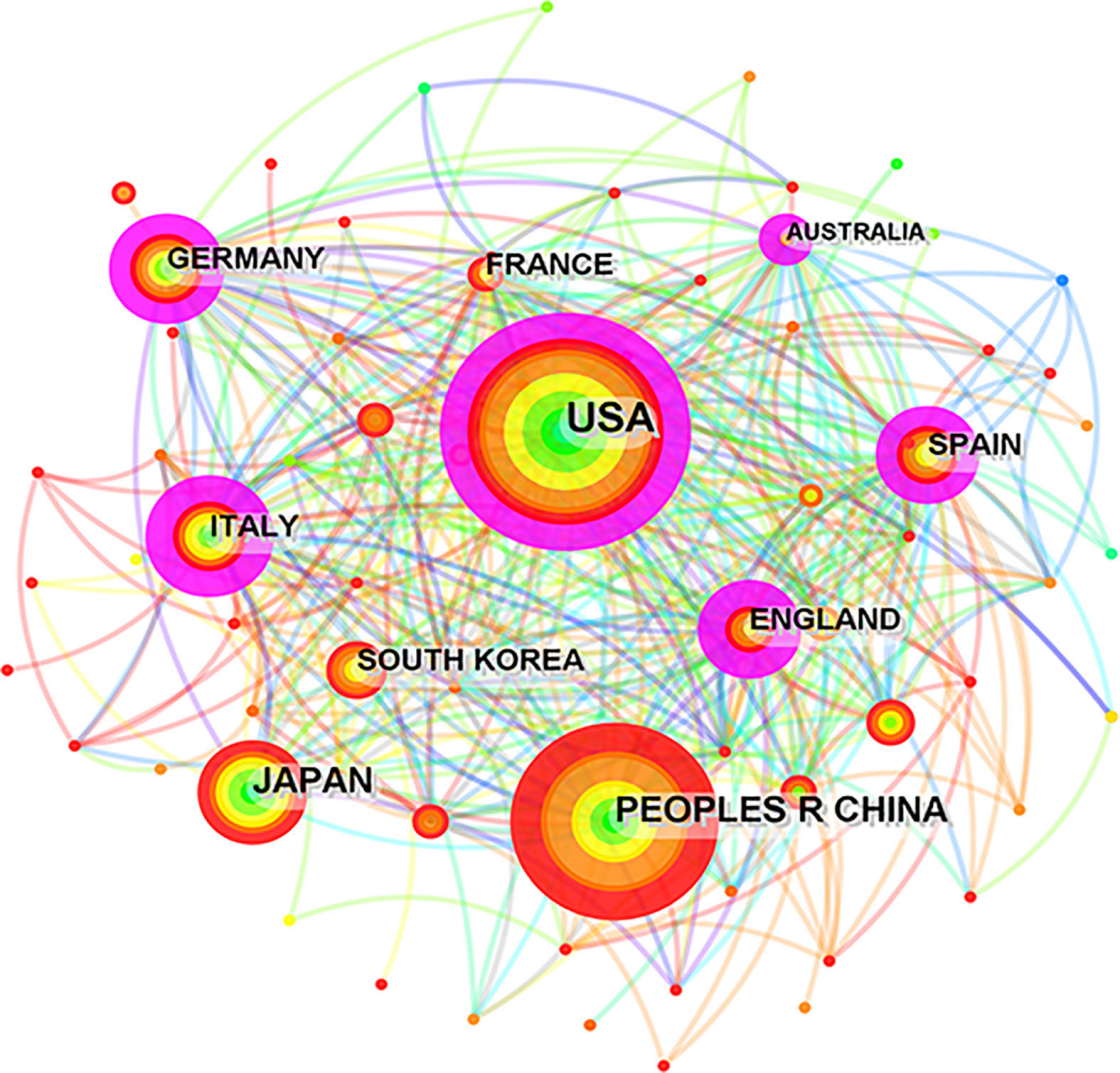
CiteSpace network map of the collaboration analysis of the studies on inflammation and fibrogenesis MAFLD among countries/ regions in 2000-2022, the top 10 countries/ regions are shown in the picture. The size of the concentric circle is positively related with the number of articles published by each country and area, the fuchsia ring indicates a node with a centrality value greater than 0.1.

At the same time, we analyzed cooperative relationships between these countries and regions *via* CiteSpace. As shown in **Figure 3**, the size of the concentric circle is positively related to the number of articles published by each country and region; the fuchsia ring indicates a node with a centrality value greater than 0.1, signifying its close relationship with other nodes. We found that 6 of the top 10 fruitful countries worked closely with others. Among them, China ranked second in terms of the number of published articles, but its international cooperation with other countries needs to be strengthened.

Moreover, we analyzed the contribution of global institutions. The most yielding institutions are shown in **Table 1**. Interestingly, among the top 10 productive institutions, 7 were from the USA, 2 came from China, and 1 was from Italy. The University of California San Diego published the most articles (74 articles), followed by Duke University (*n* = 43), Shanghai Jiao Tong University (*n* = 41), Mayo University (*n* = 37), and Harvard Medical School (*n* = 35).

### Analysis of journals

Over the past 23 years, 606 scholarly journals have published a total number of 2,348 original articles. We used VOSviewer to show journal influence. The top 10 most cited journals related to the topic of inflammation and fibrogenesis in MAFLD are presented in **Table 2**. According to the analysis, hepatology publications had the most publications (141 papers) and the most citations (22934) during the past 23 years, followed by those in *Gastroenterology* (9,737), *Journal of Hepatology* (6191), *Plos One* (3,941), and *New England Journal of Medicine* (3,421). In addition, eight journals were in the Q1 Journal Citation Reports (JCR) division, indicating their high academic standing. Notably, seven of these journals are from the USA, and the remaining three are from the Netherlands and the UK, with all of them being developed countries and therefore providing an important platform for the research development in this field.

**Table 2:** The top 10 most active journals in research of inflammation and fibrosis in MAFLD (sorted by total citation) from 2000 to 2022.

Rank	Journal	Frequency	Total citations	Average citation per paper	IF (2021)	JCR	Country
1	*Hepatology*	141	22,934	162.65	17.298	Q1	USA
2	*Gastroenterology*	30	9,737	324.57	33.883	Q1	USA
3	*Journal of Hepatology*	65	6,191	95.25	30.083	Q1	Netherlands
4	*Plos One*	85	3,941	46.36	3.752	Q2	USA
5	*New England Journal of Medicine*	3	3,421	1,140.33	176.082	Q1	USA
6	*Scientific Reports*	78	2,323	29.78	4.997	Q2	UK
7	*American Journal of Physiology-gastrointestinal and Liver Physiology*	37	1,828	49.41	12.045	Q1	USA
8	*Alimentary Pharmacology & Therapeutics*	24	1,632	68	9.524	Q1	UK
9	*American Journal of Gastroenterology*	12	1,626	135.5	12.045	Q1	USA
10	*Clinical Gastroenterology and Hepatology*	18	1,277	70.94	13.576	Q1	USA

### Analysis of authors

**Figure 4:**
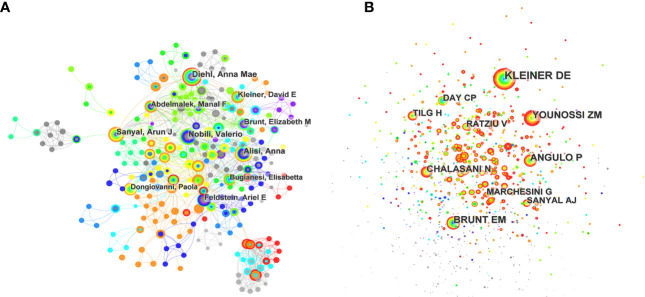
**(A)** CiteSpace visualization map of the top 10 authors. Each circle represents an author, and a link between two circles means a collaboration between each other. **(B)** CiteSpace visualization map of the top co-cited authors involved in inflammation and fibrogenesis in MAFLD research.

The top 10 most productive authors in this field are shown in **Figure 4A** and **Table 3**. Diehl AM from the Department of Gastroenterology of Duke University published 25 articles in this field and was ranked first, followed by Nobili V, Alisi A, Sanyal AJ, and Feldstein AE. She seemed interested in the connection between the Hedgehog (Hh) pathway and MAFLD (18–20) and carried out an in-depth study in this field. Furthermore, she took part in a clinical research discussing the efficacy of pioglitazone versus vitamin E versus placebo in non-diabetic patients with NASH, showing that vitamin E was a potential treatment well accepted with its high citation rate (21). Furthermore, she discussed the relationship between MAFLD and different reproductive life cycles, such as puberty and menopause (22, 23). The second one was Nobili V, from the Department of Pediatrics of Sapienza university of Rome, who has published 23 articles in this field and made great contributions to the field of children with MAFLD. His most cited article published in *Hepatology* studied lifestyle intervention and antioxidant therapy in children with MAFLD (24). Several articles suggested that various genetic mutations were associated with MAFLD, such as the PCSK7 gene variation (25) and the I148M patatin-like phospholipase domain-containing 3 gene mutation (26). Interestingly, Alisi A, from the same institution as Nobili V, published 23 articles and was ranked third. Most of her works were cooperated with Dr. Nobili V, showing their close cooperation and strong scientific research ability.

**Table 3:** The top 10 productive authors and co-cited authors in research of inflammation and fibrosis in MAFLD from 2000 to 2022.

Rank	Author	Count	Centrality	Rank	Co-cited author	Citation	Centrality
1	Diehl AM	25	0.01	1	Kleiner DE	949	0.02
2	Nobili V	23	0	2	Angulo P	585	0.06
3	Alisi A	23	0.01	3	Younossi ZM	584	0.03
4	Sanyal AJ	21	0.03	4	Brunt EM	514	0.03
5	Feldstein AE	19	0.03	5	Chalasani N	407	0.06
6	Brunt EM	17	0.02	6	Tilg H	317	0.06
7	Abdelmalek MF	17	0.04	7	Sanyal AJ	311	0.05
8	Kleiner DE	17	0.01	8	Marchesini G	302	0.04
9	Dongiovanni P	16	0	9	Ratziu V	291	0.04
10	Bugianesi E	16	0.04	10	Day CP	288	0.05

Co-cited authors are those whose works were cited in more than one study at the same time. The network visualization map for the co-cited authors is shown in **Figure 4B** and **Table 3**. Kleiner DE ranked first with a total citation of 949 times, followed by Angulo P, Younossi ZM, Brunt EM, and Chalasani N. Among the top 10 most co-cited authors, 3 were the top 10 productive authors, and 4 were the first authors of the most cited articles of derived papers shown in **Table 4**. In general, the low centrality value indicated a lack of cooperation among them.

**Table 4:** The top 10 most cited articles in research of inflammation and fibrosis in MAFLD from 2000 to 2022.

Rank	Title	First Author	Journal	Year	Citations	DOI
1	Design and validation of a histological scoring system for nonalcoholic fatty liver disease	Kleiner, DE	*Hepatology*	2005	6,900	10.1002/hep.20701
2	Pioglitazone, Vitamin E, or Placebo for Nonalcoholic Steatohepatitis.	Sanyal, AJ	*New England Journal of Medicine*	2010	2,057	10.1056/NEJMoa0907929
3	Liver fibrosis, but no other histologic features, is associated with long-term outcomes of patients with nonalcoholic fatty liver disease	Angulo, P	*Gastroenterology*	2015	1,627	10.1053/j.gastro.2015.04.043
4	Sampling variability of liver biopsy in nonalcoholic fatty liver disease	Ratziu, V	*Gastroenterology*	2005	1,399	10.1053/j.gastro.2005.03.084
5	A placebo-controlled trial of pioglitazone in subjects with nonalcoholic steatohepatitis	Belfort, R	*New England Journal of Medicine*	2006	1,248	10.1056/NEJMoa060326
6	Hepatocyte apoptosis and Fas expression are prominent features of human nonalcoholic steatohepatitis	Feldstein, AE	*Gastroenterology*	2003	781	10.1016/S0016-5085(03)00907-7
7	Elafibranor, an agonist of the peroxisome proliferator-activated receptor-alpha and -delta, induces resolution of nonalcoholic steatohepatitis without fibrosis worsening	Ratziu, V	*Gastroenterology*	2016	642	10.1053/j.gastro.2016.01.038
8	Efficacy and safety of the Farnesoid X receptor agonist obeticholic acid in patients with type 2 diabetes and nonalcoholic fatty liver disease	Mudaliar, S	*Gastroenterology*	2013	630	10.1053/j.gastro.2013.05.042
9	A pilot study of ploglitazone treatment for nonalcoholic steatohepatitis	Promrat, K	*Hepatology*	2004	527	10.1002/hep.20012
10	Toll-like receptor 9 promotes steatohepatitis by induction of interleukin-1 beta in mice	Miura, K	*Gastroenterology*	2010	523	10.1053/j.gastro.2010.03.052

### Analysis of document citation

Citation analysis is a reliable indicator for assessing the quality of articles, the results were derived from WoSCC-SCIE, and the top 10 most cited articles are shown in **Table 4**. The article published in *Hepatology* in 2005 ranked first. It presented a scoring system to assess a large range of histological features of NAFLD for pediatric and adult NAFLD (27). The next one was published in the *New England Journal of Medicine* in 2010, which was a clinical study discussing the efficacy of pioglitazone and vitamin E for the treatment of NASH in adults without diabetes. It was conducted in cooperation with Diehl AM, who ranked first in the number of published articles (21). The third one also focused on histological features and approved the crucial role of fibrosis stage in managing and monitoring in NAFLD patients (28). Among the top 10 most cited articles, 6 were from *Gastroenterology*, 2 were from *Hepatology*, and 2 were from the *New England Journal of Medicine*. It is worth noting that all of the above three journals were top-cited, enhancing the reliability of the results shown in **Table 2**.

### Analysis of co-cited references and clustered network

**Figure 5:**
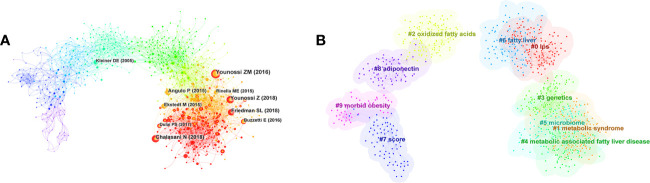
**(A)** CiteSpace co-citation map of references involved in inflammation and fibrogenesis in MAFLD. The first authors of the top 10 most co-cited references are presented. **(B)** Clustered networks of the co-citation analysis of the investigated reference and the 2348 citing articles via CiteSpace. The top 10 largest clusters are shown.

Co-cited references are two or more references cited by another paper or more papers simultaneously. From 2000 to 2022, a total of 2,348 articles and their 53,816 references retrieved from WoSCC-SCIE were analyzed by CiteSpace, the first authors of the top 10 most co-cited references are presented in **Figure 5A**, and the summary of the top 10 most co-cited references is shown in **Table 5**. Interestingly, it seems to be well-accepted that the fibrosis stage of patients with MAFLD is an independent factor for the long-term outcomes, such as mortality, liver transplantation, and liver-related events (28, 29). In brief, the most cited references shown in **Table 5** made great contributions to the development of inflammation and fibrogenesis in the MAFLD scientific community and were the most recognized papers in this field.

**Table 5:** The top 10 high-cited references in research of inflammation and fibrosis in MAFLD from 2000 to 2022.

Rank	Title	First Author	Year	Journal	Citations	DOI
1	Global epidemiology of nonalcoholic fatty liver disease—Meta- analytic assessment of prevalence, incidence, and outcomes	Younossi ZM	2016	*Hepatology*	215	DOI 10.1002/hep.28431
2	The diagnosis and management of nonalcoholic fatty liver disease: Practice guidance from the American Association for the Study of Liver Diseases	Chalasani N	2018	*Hepatology*	163	DOI 10.1002/hep.29367
3	Global burden of NAFLD and NASH: Trends, predictions, risk factors and prevention	Younossi ZM	2018	*Nat Rev Gastro Hepat*	158	DOI 10.1038/nrgastro.2017.109
4	Mechanisms of NAFLD development and therapeutic strategies	Friedman SL	2018	*Nat Med*	139	DOI 10.1038/s41591-018-0104-9
5	Liver fibrosis, but no other histologic features, is associated with long-term outcomes of patients with nonalcoholic fatty liver disease	Angulo P	2015	*Gastroenterology*	110	DOI 10.1053/j.gastro.2015.04.043
6	The multiple-hit pathogenesis of non-alcoholic fatty liver disease (NAFLD)	Buzzetti E	2016	*Metabolism*	84	DOI 10.1016/j.metabol.2015.12.012
7	Fibrosis stage is the strongest predictor for disease-specific mortality in NAFLD after up to 33 years of follow-up	Ekstedt M	2015	*Hepatology*	84	DOI 10.1002/hep.27368
8	Design and validation of a histological scoring system for nonalcoholic fatty liver disease	Kleiner DE	2005	*Hepatology*	75	DOI 10.1002/hep.20701
9	Nonalcoholic fatty liver disease: A systematic review	Rinella ME	2015	*JAMA—J Am Med Assoc*	66	DOI 10.1001/jama.2015.5370
10	Increased risk of mortality by fibrosis stage in nonalcoholic fatty liver disease: Systematic review and meta-analysis	Dulai PS	2017	*Hepatology*	64	DOI 10.1002/hep.29085

**Figure 6:**
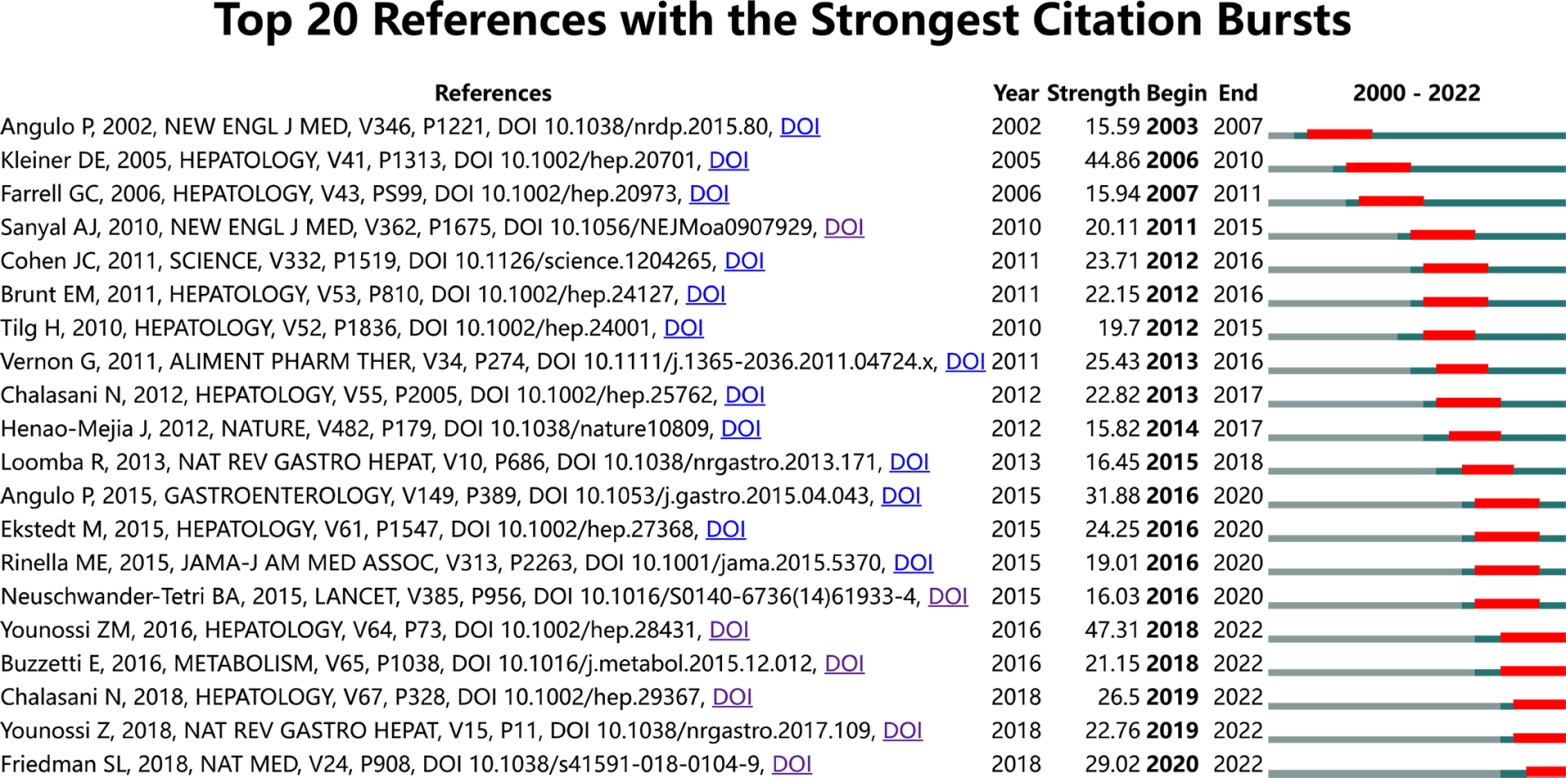
Top 20 references with the strongest citation bursts.

In addition, we also analyzed the strong citation burst of references in this topic. The references of the top 20 references with the strongest citation bursts are shown in **Figure 6**. “Year” refers to the publication date, “Begin” refers to the first citation, and “End” refers to the last citation. We found that the two papers with 4 years’ duration were all focused on seeking effective treatment methods for MAFLD, indicating experts’ concern for this disease (21, 30). The strongest citation reference was a meta-analysis review published by *Hepatology* in 2016, which is still widely cited and was the top high-cited reference, discussing the epidemiology of NAFLD, including prevalence, incidence, and long-term outcomes (2). Five other pieces of literature still widely cited described the mechanism, diagnosis, treatment, and global burden of MAFLD (31–33).

The map of the co-citation cluster according to keywords generated from the references of 2,348 citing articles by CiteSpace is shown in **Figure 5B**. The clustering modularity *Q* and the mean silhouette value were 0.6833 and 0.8729, respectively, demonstrating a credible structure and clustering results. Furthermore, the number of cluster labels is inversely proportional to the number of articles included in each cluster. Thus, the “#0 LPS” cluster contains the most papers, while the “#9 morbid obesity” cluster contains the fewest. The summary of clusters is listed in **Table 6**.

**Table 6:** Summary of 10 clusters.

Cluster ID	Top Term	Size	Silhouette
0	LPS	142	0.841
1	Metabolic syndrome	126	0.825
2	Oxidized fatty acids	121	0.907
3	Genetics	116	0.877
4	Metabolic associated fatty liver disease	106	0.846
5	Microbiome	91	0.829
6	Fatty liver	90	0.902
7	Score	72	0.961
8	Adiponectin	70	0.896
9	Morbid obesity	47	0.92

### Analysis of the research trend and burst detection

**Figure 7:**
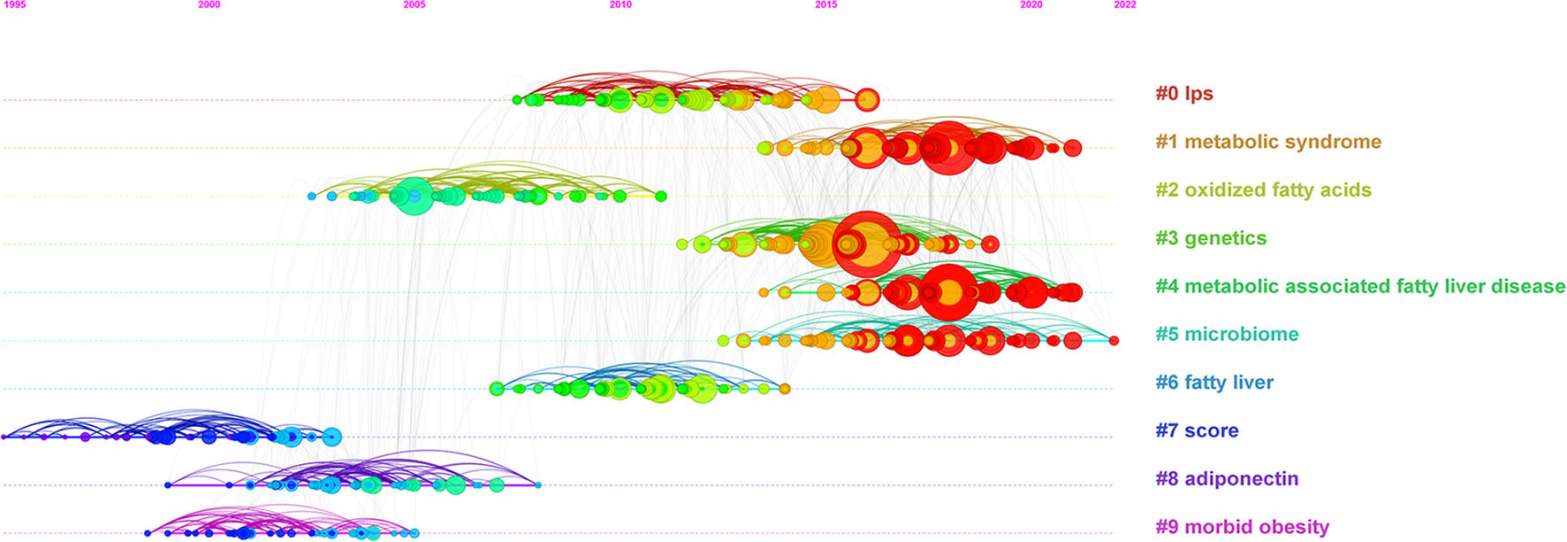
A timeline view of the top largest clusters of citing articles.

In order to analyze the change in research hotspots and trends, a timeline view is displayed in **Figure 7**. We found that early research concentrated on “#8 adiponectin” and “#9 morbid obesity”, interim studies concentrated on “#0 LPS”, “#2 oxidized fatty acids”, and “#6 fatty liver”, whereas current studies concentrated on “#1 metabolic syndrome (MS),” “#3 genetics”, and “#5 microbiome”, indicating changes in research hotspots.

**Figure 8:**
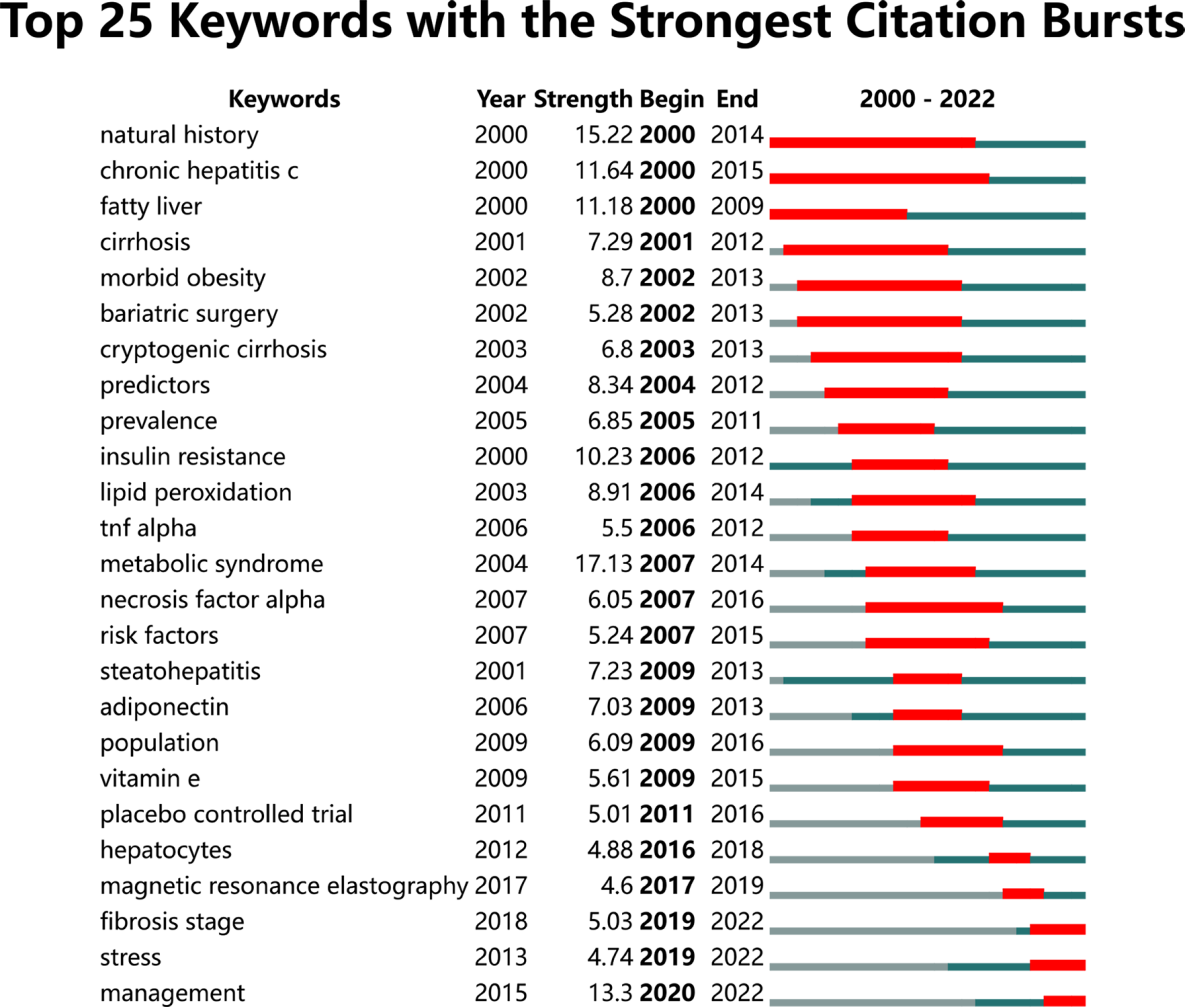
Top 25 keywords with the strongest citation bursts.

Keyword burst detection is another effective way to explore research hotspots. **Figure 8** shows the top 25 keywords with the strongest citation bursts on research from 2000 to 2022. Among the identified keyword bursts, chronic hepatitis C began in 2000 and ended in 2015 with the longest lasting time, which was associated with liver steatosis and was a risk factor for MAFLD. Moreover, three keyword bursts continue to last until the end of 2022, including management, stress, and fibrosis stage.

## Discussion

In this bibliometric analysis study, we found 2,348 articles regarding inflammation and fibrogenesis in MAFLD from 2000 to 2022 in the WoSCC-SCIE. With the help of VOSviewer and CiteSpace software, our study analyzed publication trends about inflammation and fibrogenesis in MAFLD from all aspects to shed light on researchers interested in this field.

Between 2000 and 2013, less than 100 articles concerning inflammation and fibrogenesis in MAFLD were published per year globally. However, the number of papers has increased rapidly since 2018, which was associated with the renaming of NAFLD and its high morbidity, indicating that the research on inflammation and fibrogenesis in MAFLD has caught the attention of researchers worldwide. **Table 1** shows that the USA played a vital role in the research on inflammation and fibrogenesis in MAFLD. Notably, with the development of China’s medical and scientific research capability, the number of articles published in China has rapidly increased and firstly surpassed the USA in 2021. Moreover, with the development of globalization, international cooperation has become a new trend that benefits the output of high-quality research. The collaborations between countries/regions are shown in **Figure 3**, most (*n* = 6) of the top 10 productive countries/regions cooperated closely with other countries, but China greatly needs to strengthen its cooperation with other countries.

Remarkably, 7 of the top 10 productive institutions and 7 of the top 10 most cited journals are from the USA, reasonably explaining that the USA occupies the main contribution on total numbers of published papers. Therefore, these results demonstrated that the USA played a dominant role in the world’s academic activities. However, China may be a rising star in the next few years, considering the number of papers published in this field and its rising trends.

The timeline view in **Figure 7** indicates the evolution of the research trend. We found that early research concentrated on adiponectin and morbid obesity. Adiponectin is an adipocyte-specific secretory protein that plays a pivotal role in glycolipid metabolism (34) and extracellular matrix (ECM) metabolism (35) and has made a great contribution to preventing the liver from steatosis, inflammation, and fibrosis (36, 37). It has been reported that MAFLD patients have significantly lower plasma adiponectin levels (38). Multivariate regression analysis identifies decreased adiponectin as an independent risk factor of hepatic steatosis (37). Furthermore, abundant clinical and basic studies have illustrated that adiponectin agonists are therapeutic targets for NAFLD therapy (39, 40). Morbid obesity was defined as a body mass index (BMI) of at least 40 kg/m^2^. Many researchers suggest that Roux-en-Y gastric bypass (RYGB) surgery is a decent curative method in morbid obesity patients with MAFLD to improve inflammation and fibrosis and then inhibit malignant progression to NASH (41–43). Subsequently, interim research mainly focused on identifying novel markers and cellular and molecular mechanisms in MAFLD (44, 45).

With the deepening study in this field, there is increasing evidence to show that MS is an important risk factor in MAFLD, vividly illustrated by the renaming of NAFLD to MAFLD. MS mainly refers to hyperglycemia, abdominal obesity, and dyslipidemia. A systematic review attempted to estimate the prevalence of MAFLD among patients with T2DM and found that the prevalence of MAFLD was 55.5% among 49,419 T2DM patients (46). Another research suggested that more than 90% of obese patients with T2DM also have MAFLD (47), indicating that MAFLD is strongly associated with hyperglycemia. Moreover, studies among T2DM participants have demonstrated that plasma glucose level is positively correlated with the risk of developing advanced liver disease (HCC) (48). Furthermore, glycemic variability, except for hyperinsulinemia and hyperglycemia, is an independent predictive factor for the progression of hepatic fibrosis in MAFLD (49). Considering that there is no approved drug for NASH to date, lifestyle interventions and combinations of drugs, which can effectively regulate glucose and lipid metabolism and reduce liver inflammation and fibrosis, might be a beneficial option to curb the deteriorated progression of MAFLD. Even new drug development focuses on the restitution of metabolic derangements and halting inflammatory and fibrogenic pathways, showing that MS plays an important role in the development of MAFLD (50).

Furthermore, it has been reported that genetic factors took part in the progression of MAFLD due to the upgrading and improvement of new genomic and proteomic technologies (51), and many risk variants of the NAFLD population were identified by a genome-wide association study (GWAS) (52). Currently, at least five variants in different genes are associated with the development and progression of MAFLD, namely, PNPLA3, TM6SF2, GCKR, MBOAT7, and HSD17B13 (53–55). Some of them are associated with an increased risk of T2DM. Others are associated with the risk of developing obesity (56, 57), indicating that MAFLD might have shared mechanisms that are involved in the pathogenesis of T2DM and obesity, emphasizing the importance of MS in MAFLD, and supporting the renaming of NAFLD to MAFLD (55).

Gut microbiota is another research hotspot now. Many studies showed that microbiota might improve or aggravate MAFLD through multiple mechanisms (58–60), including changing the permeability of the intestine (61), altering the expression of genes involved in the *de novo* lipogenesis (62), and regulating choline (63) and bile acid metabolism (64). The most extensively studied microbial molecule is lipopolysaccharide (LPS), which is produced by Gram-negative bacteria. It has been reported that systemic LPS concentration was significantly elevated in rats treated with HFD and high-sucrose diet (65) and in MAFLD patients (66). In related animal studies, the effect of LPS on the development of MAFLD has also been shown in mice injected with LPS and mice lacking toll-like receptor 4 (TLR4) (67, 68). A number of studies investigated that modulation of the gut microbiota may be a potential therapeutic target for MAFLD, including using antibiotics, prebiotics, and probiotics (69).

Generally speaking, **Figure 7** demonstrates that the research hotspots have shifted their direction to MS, genetics, and the microbiome in the study of inflammation and fibrosis in MAFLD.

Another effective method of reflecting the transition of hotspots in an academic area is to use keyword bursts. The top 25 keywords with the strongest citation bursts are shown in **Figure 8**. Among them, three keywords continue to last by the end of 2022. The first one is management. The related research with the highest citation was published in *Hepatology* in 2019, elucidating that gut microbiota profile and systemic inflammatory response in NAFLD were closely related, further promoting the process of HCC (70). Some of the works concerned the relationship between MAFLD and common chronic diseases (71, 72), and most publications explored effective methods to curb the development of MAFLD and its complications (73–76). The second one was fibrosis stage, a valuable parameter to predict all-cause and liver disease-related mortality in MAFLD (77). The related articles mainly focused on seeking potential ways to restrain the adverse progression of fibrosis stage and thus improve the prognosis (78–80). For example, Khurana and Wang attempted to find an invasive method that is helpful for early diagnosis, distinguishing disease staging, and giving personalized treatments (81, 82). The third keyword burst, which started in 2019, was stress. Articles related to this keyword refer to a metabolic stress state relevant to the dysfunction of mitochondria and ER and describe its potential mechanisms that have a significant impact on the progress of MAFLD. Zhang et al. suggested that impaired mitophagy, which may lead to the accumulation of excessive ROS production and oxidative stress, triggered hepatic NLRP3 inflammasome activation during the progress of MAFLD (83). In addition, another research noted that down-regulating the NLRP3/NF-κB signaling pathway can attenuate inflammation in mouse liver (84). Meanwhile, the authors identified apoptosis signal-regulating kinase 1 (ASK1) as a suppressor of NASH and fibrosis formation *via* ASK1 knockout experiments. All of them reminded us that inflammation and fibrosis are key factors in the development of MAFLD and drugs that act on them may have potential as a clinical treatment to prevent MAFLD in humans.

Of note, magnetic resonance elastography (MRE), a keyword that lasted from 2017 to 2019, is a non-invasive evaluation to distinguish healthy people from those with NAFLD and assess the degree of fibrosis to discriminate simple steatosis from NASH (85), showing that colleagues are actively looking for non-invasive methods to determine the severity of NAFLD, and thus ensure timely and optimal treatment.

Notably, the number of publications focusing on inflammation and fibrosis in MAFLD increased rapidly. However, previous studies on inflammation and fibrogenesis in MAFLD still have certain limitations, and there is much to be improved in the future.

1. It is urgent to identify more valuable non-invasive biomarkers to meet the need to accurately stage the progression of MAFLD, make a definitive diagnosis as soon as possible, and provide the patients with timely and effective treatment.
2. More research on developing novel agents targeting hepatic inflammation and fibrosis are needed as there are no approved drugs for NASH.
3. There are numerous basic studies, but few can be applied to the clinic. The use of non-human primate models rather than rodents in mechanistic investigations would likely allow for a higher chance of translating basic discoveries into clinical practice.

Our study has some limitations. First, we only analyzed data exported from WoSCC-SCIE to undertake relevant analysis, which may result in selection bias. Second, this study excluded non-English literature, and some high-quality non-English literature was excluded. Third, VOSviewer and CiteSpace have certain defects that may output discredited results. Nevertheless, our study still provides significant information and insights for researchers interested in this field.

## Conclusion

From 2000 to 2022, the number of articles focusing on inflammation and fibrosis in MAFLD increased rapidly, especially in the last 5 years. The USA played a vital role in the development of this topic regarding the number of publications, international cooperation with other countries, and achievements of authors. However, China may be a rising star in this research field with its increasing trend in the number of publications and its huge population. Current research mainly focuses on MS, genetics, and microbiome. Considering the urgent and emergent situation of inflammation and fibrosis in MAFLD, more studies are needed to focus on developing novel drugs and identifying non-invasive biomarkers. In short, our study provides a comprehensive overview of this discipline, which could more precisely direct scholars in future research and provide valuable guidance for clinical diagnosis, appropriate treatment, and individualized prevention.

## Data availability statement

The original contributions presented in the study are included in the article/supplementary material. Further inquiries can be directed to the corresponding author.

## Author contributions

All the authors have contributed significantly. HL designed the study. KL and YC wrote the manuscript. KL, YC, SF, SW, and ZW collected and analyzed the data, participated in discussion. SF and HL supervised the study and corrected the manuscript. All authors contributed to the article and approved the submitted version.
